# Factors Influencing Interest in Urology Among Osteopathic and Allopathic Medical Students: A Single-Institution Cross-Sectional Survey

**DOI:** 10.7759/cureus.107827

**Published:** 2026-04-27

**Authors:** Emmaline Woodworth, Carter Sheppard, Ryan Wong, Harvey N Mayrovitz

**Affiliations:** 1 College of Osteopathic Medicine, Nova Southeastern University Dr. Kiran C. Patel College of Osteopathic Medicine, Davie, USA; 2 College of Allopathic Medicine, Nova Southeastern University Dr. Kiran C. Patel College of Allopathic Medicine, Davie, USA; 3 Medical Education, Nova Southeastern University Dr. Kiran C. Patel College of Allopathic Medicine, Davie, USA

**Keywords:** allopathic medical education, cross-sectional survey, medical student mentorship, osteopathic medical education, residency match disparities, specialty exposure, surgical specialty interest, urology match, urology residency

## Abstract

Background

Following the transition to a single-accreditation residency system, Doctor of Osteopathic Medicine (DO) and Doctor of Allopathic Medicine (MD) students now apply for the same urology residency positions. Despite this unification, disparities in match rates persist, and factors influencing student interest in and the perceived feasibility of pursuing urology remain unclear.

Objective

To compare and explore differences in perceptions of pursuing urology between DO and MD medical students at a single institution, with primary outcomes including perceived attainability of a urology career, level of specialty exposure, and access to urology-specific resources.

Methods

A 20-question cross-sectional survey was distributed to all enrolled DO (n = 1,636) and MD (n = 220) students at Nova Southeastern University in fall 2025. Participation was voluntary and anonymous. Survey items assessed specialty interest, exposure, resource access, and perceived feasibility of pursuing urology. Fisher’s exact tests were used for DO-MD comparisons, and chi-square goodness-of-fit tests were used for within-group DO analyses. This study was conducted and reported in accordance with Strengthening the Reporting of Observational Studies in Epidemiology (STROBE) guidelines.

Results

A total of 113 responses were analyzed: 81 DO and 32 MD students. Among students interested in urology (28 DO, 11 MD), 32.1% of DO students (9/28) and 54.5% of MD students (6/11) perceived the specialty as a realistic career goal; this difference was not statistically significant (Fisher’s exact p = 0.27). When all DO responses were collapsed into “Yes” versus “Not Yes,” only 16 of 81 DO students (19.8%) endorsed urology as realistic, whereas 65 of 81 (80.2%) responded “No” or “Not sure” (p < 0.001). Exposure to urology during medical school was reported by 30 of 81 DO students (37.0%) and 16 of 32 MD students (50.0%) (p = 0.23). Overall, 24 of 81 DO students (29.6%) reported no exposure to urology before or during medical school. MD students consistently reported greater access to mentorship and specialty-specific resources, but these differences did not reach statistical significance. Students from both programs cited surgical variety and lifestyle as key motivations for pursuing urology and similarly valued degree-concordant mentorship.

Conclusions

DO students in this sample were less likely to perceive urology as an attainable career and reported lower levels of specialty exposure and access to urology-specific resources. Although between-group differences did not reach statistical significance, the observed patterns raise the possibility that differences in exposure and resource availability, rather than motivation alone, may contribute to these perceptions. These findings are exploratory and hypothesis-generating and warrant further investigation in larger, multi-institutional cohorts.

## Introduction

In 2014, the Accreditation Council for Graduate Medical Education (ACGME), the American Osteopathic Association (AOA), and the American Association of Colleges of Osteopathic Medicine (AACOM) reached an agreement to unite the residency match system for students who obtained Doctor of Osteopathic Medicine (DO) and Doctor of Allopathic Medicine (MD) degrees [1]. This single-accreditation system allowed DO and MD students to apply for all available residency positions, regardless of their degree program. The urology residency match system, separately governed by the American Urological Association (AUA), underwent a similar merger with the AOA in 2021 [2-4]. Prior to the merger, 11 urology residency programs were historically osteopathic. With the unification of the match, osteopathic applicants faced the loss of designated osteopathic positions but gained the opportunity to apply for more than 400 positions alongside their MD colleagues [2-5].

According to the AOA, more than 25% of current US medical students earn their medical degrees from colleges of osteopathic medicine, and 99% of seniors who obtained the DO degree secured residency placement in 2025 [6]. Notably, residency match data from the AUA indicate that only 10.8% of urology applicants were senior DO students or previous DO graduates [5]. Among senior DO applicants, only 57% successfully matched, compared with 86% of senior MD applicants. A similar disparity was observed among prior graduates, with match rates of 29% for DOs versus 56% for MDs [5].

The explanation for this disparity is multifactorial and complex. First, over 75% of DO schools lack affiliation with a home urology program [7]. This limits exposure to urology during clinical years, narrows access to urology-specific research opportunities, and creates a barrier to obtaining letters of recommendation for residency applications [8]. Additionally, only 2.5% of current practicing urologists are osteopathic physicians, limiting mentorship opportunities within the degree program [2]. Lastly, medical students often rely on peer support and advice from upperclassmen when navigating the residency application process. With only 56 DO seniors and graduates applying for urology residency positions in 2025, opportunities for peer support are sparse [5].

Nova Southeastern University is one of only three universities in the United States with both MD and DO medical schools under one roof [9]. This allows the Nova Southeastern University collective medical student body to serve as a unique study population for comparing the attitudes and beliefs of DO and MD students, while limiting confounding factors such as geographic differences and university resources.

To the best of our knowledge, no prior studies have directly compared factors influencing DO and MD students’ interest in pursuing urology. The objective of this cross-sectional survey study is to explore and compare differences in attitudes, specialty exposure, and perceived access to urology-related resources between DO and MD students at a single institution. Given the limited existing data, this study is intended to be exploratory and hypothesis-generating.

## Materials and methods

This cross-sectional survey study was conducted at Nova Southeastern University, approved and marked exempt by the Institutional Review Board (NSU IRB Study: 2025-495), and reported in accordance with the Strengthening the Reporting of Observational Studies in Epidemiology (STROBE) guidelines.

A 20-question survey was developed using Research Electronic Data Capture (REDCap) based on commonly described factors influencing medical student specialty selection. Draft questions were reviewed by faculty with experience in medical education to ensure content relevance and clarity. The survey was pilot-tested with a small group of medical students not included in the final study population, and minor revisions were made prior to distribution. The final survey included 18 multiple-choice questions with categorical response options and two open-ended questions. Survey data were collected electronically via REDCap, and all responses were self-reported by participants.

The survey was designed to assess DO and MD students’ experiences and perceptions regarding pursuing urology as a specialty. The first three questions collected demographic information, including degree program, medical school year, and gender identity. The next four questions assessed respondents’ interest in and knowledge of the specialty. The remaining 13 questions evaluated attitudes and perceptions regarding program resources that may influence interest in urology. Primary outcome variables included perceived feasibility of pursuing urology, exposure to the specialty, and access to urology-related resources. Secondary variables included mentorship preferences and factors influencing interest in urology, assessed through both categorical and open-ended responses. Survey questions and the complete item-level response distribution are provided in the Appendix 1.

The survey was disseminated via institutional email listservs to all first-, second-, third-, and fourth-year students enrolled at Nova Southeastern University in both the Dr. Kiran C. Patel College of Osteopathic Medicine and the Dr. Kiran C. Patel College of Allopathic Medicine, representing a census sampling approach with no additional inclusion or exclusion criteria beyond enrollment status. A total of 1,636 osteopathic students across two campuses, Davie and Tampa, Florida, and 220 allopathic students were invited to participate. Responses were voluntary and anonymous. The survey was distributed to osteopathic students on September 22, 2025, and to allopathic students on October 8, 2025. To minimize nonresponse bias, a follow-up reminder was sent to both groups on October 29, 2025. The study period spanned September 22, 2025, to November 25, 2025. Incomplete survey responses were excluded using a complete-case analysis approach, and no imputation for missing data was performed.

Potential sources of bias include nonresponse bias due to differential response rates between groups, as well as recall and social desirability bias, which are inherent to self-reported survey data. No formal sample size calculation was performed, and all complete available responses during the study period were included in the analysis.

Descriptive statistics were used to summarize survey responses. Fisher’s exact tests were used for between-group comparisons between DO and MD students due to small sample sizes, and chi-square goodness-of-fit tests were used for within-group DO analyses. Fisher’s exact test was selected to account for small cell counts and avoid violations of chi-square assumptions, while chi-square goodness-of-fit testing was used for within-group analyses where expected frequencies were appropriate. A two-sided p-value < 0.05 was considered statistically significant. Effect sizes were reported as risk differences with corresponding 95% CIs for all between-group comparisons. Open-ended responses were reviewed and summarized descriptively to identify commonly reported reasons; no formal qualitative coding framework was applied.

## Results

A total of 113 responses were received, including 81 (71.7%) from DO students and 32 (28.3%) from MD students, yielding response rates of 5.0% and 14.5%, respectively. No partially completed surveys met inclusion criteria; therefore, no responses were excluded after submission. Among all 113 respondents, 44 (38.9%) identified as male, 68 (60.2%) identified as female, and 1 (0.9%) preferred not to disclose their gender identity. When examining respondent training stage, 99 (87.6%) respondents were in the pre-clinical years (M1: 52 (46.0%); M2: 47 (41.6%)), while 14 (12.4%) were in the clinical years (M3: 9 (8.0%); M4: 5 (4.4%)). Table 1 summarizes these features. There were no missing data among included responses.

**Table 1:** Demographic characteristics of survey respondents (n = 113). Values are presented as number (%). DO: Doctor of Osteopathic Medicine; MD: Doctor of Medicine; M1-M4: first- through fourth-year medical students. Percentages may not total 100% because of rounding.

Characteristic	Category	No. (%)
Degree program	DO	81 (71.7%)
	MD	32 (28.3%)
Gender identity	Male	44 (38.9%)
	Female	68 (60.2%)
	Non-binary	0 (0.0%)
	Prefer not to say	1 (0.9%)
Year in medical school	M1	52 (46.0%)
	M2	47 (41.6%)
	M3	9 (8.0%)
	M4	5 (4.4%)

Pursuit and perceived feasibility of urology

**Figure 1:**
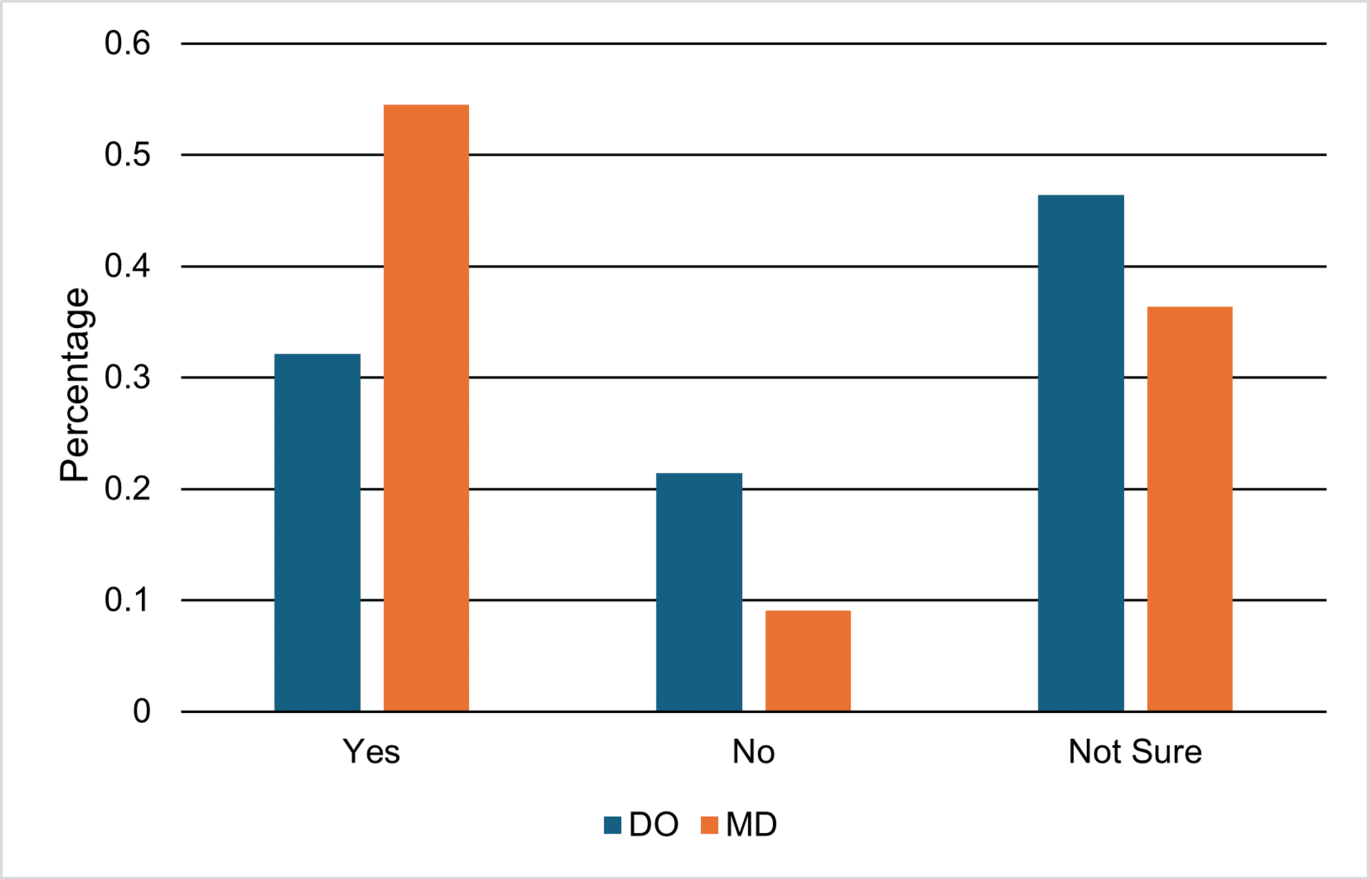
Responses regarding urology as a realistic career goal among students interested in urology. Percentages of osteopathic medical student respondents (DO) (n = 28) and allopathic medical student respondents (MD) (n = 11) who expressed interest in urology and perceived urology as a realistic career goal.

Among students who expressed interest in pursuing urology, 28 were DO students and 11 were MD students. Of these, 32.1% of DO students (9/28) and 54.5% of MD students (6/11) perceived urology as a realistic career goal (Figure 1). This difference was not statistically significant (Fisher’s exact p = 0.27; OR, 2.53; 95% CI, 0.63-10.2). When all DO responses were collapsed into “Yes” versus “Not yes,” a chi-square goodness-of-fit test demonstrated a significant difference in the distribution of responses among DO students (χ²(1, N = 81) = 29.6, p < 0.001). Only 19.8% of DO students endorsed urology as a realistic goal, whereas 80.2% responded either “No” or “Not sure,” suggesting potential perceived barriers or uncertainty regarding the feasibility of pursuing urology.

When assessing perceived feasibility by training stage, students were grouped into pre-clinical (M1-M2) and clinical (M3-M4) cohorts. After dichotomizing responses into “Yes” versus “Not yes,” 22.2% of pre-clinical students (22/99) and 7.1% of clinical students (1/14) reported urology as a realistic career goal, with no statistically significant difference between groups (risk difference, 15.1%; 95% CI: -5.5% to 35.7%; Fisher’s exact test, p = 0.29).

Exposure to urology

**Figure 2:**
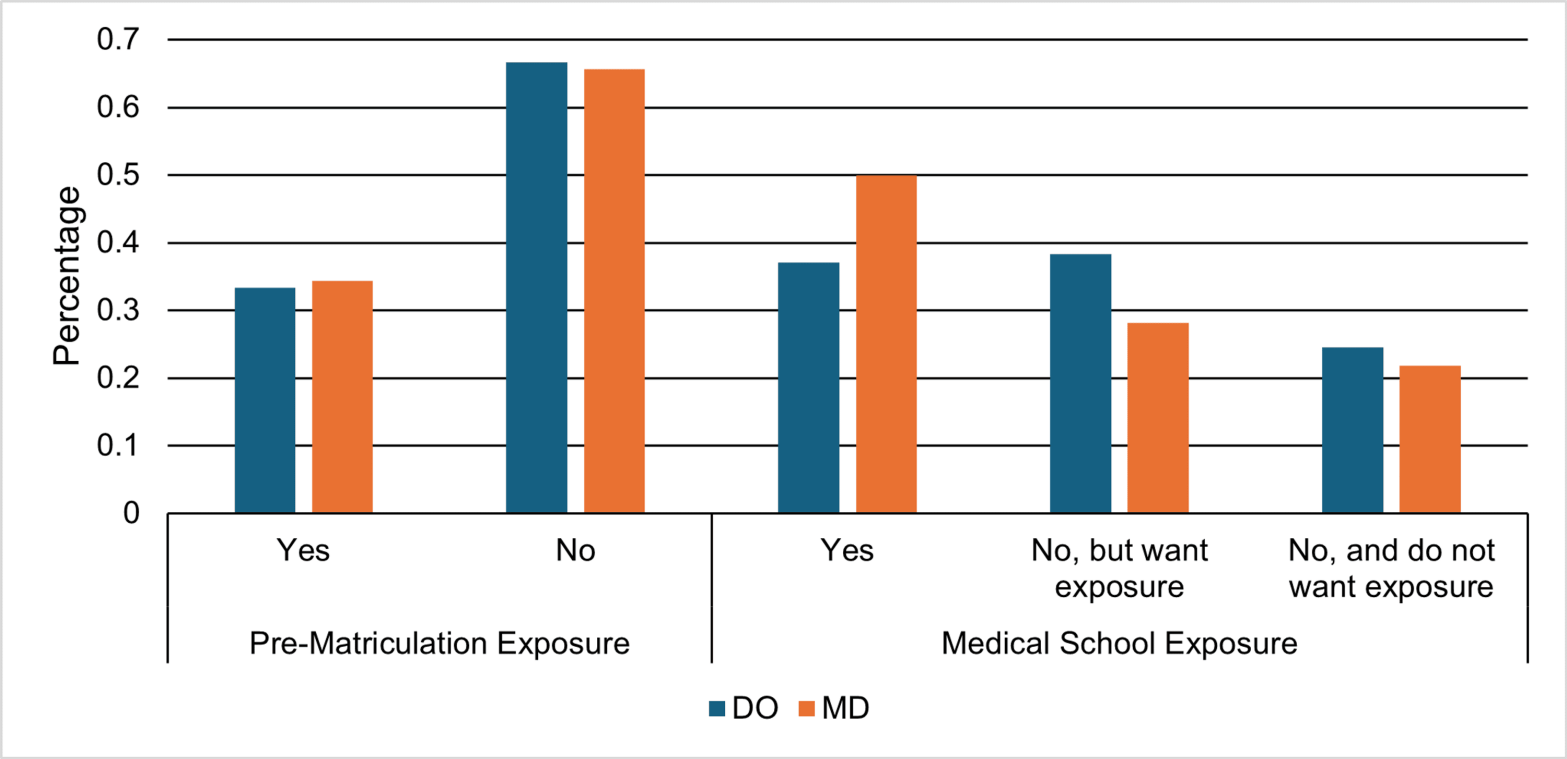
Exposure to urology before and during medical school. Percentages of osteopathic medical student respondents (DO) (n = 81) and allopathic medical student respondents (MD) (n = 32) who reported exposure to urology prior to matriculation and during medical school.

Among DO respondents, 27 (33.3%) reported exposure to urology prior to matriculation, whereas 30 (37.0%) reported exposure during medical school. Of those DO students who had not received exposure during medical school, 31 (60.8%) expressed a desire for such exposure. Overall, 24 (29.6%) DO respondents reported no exposure to urology prior to or during medical school (Figure 2). Among MD respondents, 11 (34.4%) reported exposure prior to matriculation, while 16 (50.0%) reported exposure during medical school. Of those who had not received exposure during medical school, 9 (56.3%) expressed a desire for such exposure. Overall, 11 (34.4%) MD respondents reported no exposure to urology prior to or during medical school (Figure 2). Although MD students reported greater exposure during medical school than DO students (50.0% vs 37.0%), the difference in exposure during medical school between DO and MD students was not statistically significant (risk difference, -13.0%; 95% CI: -31.5% to 5.5%; Fisher’s exact test, p = 0.23).

Urology resource availability

When evaluating awareness and availability of urology-related resources, a smaller proportion of DO students reported access to urology-related research opportunities compared with MD students (8.6% (n = 7) vs 21.9% (n = 7)); however, this difference was not statistically significant (risk difference, -13.3%; 95% CI: -27.4% to 0.8%; Fisher’s exact test, p = 0.08). Despite the presence of urology-focused student organizations in both programs, 25 DO students (30.9%) reported being unaware of such organizations, compared with 13 MD students (40.6%); this difference was not statistically significant (risk difference, -9.7%; 95% CI: -29.3% to 9.9%; Fisher’s exact test, p = 0.33).

Peer group support 

**Figure 3:**
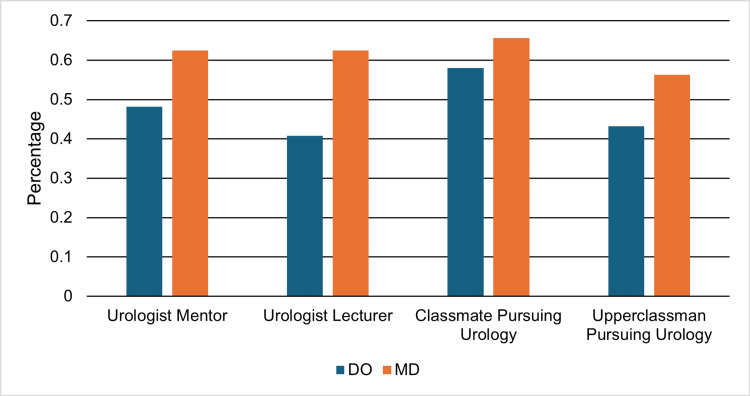
Urology-related resources. Percentages of osteopathic medical student respondents (DO) (n = 81) and allopathic medical student respondents (MD) (n = 32) who reported access to urologist mentors, urologist-led lectures, classmates interested in pursuing urology, and upperclassmen interested in pursuing urology.

MD students consistently reported greater perceived availability of urology-related mentorship and peer support. A higher proportion of MD students reported the ability to find a urologist mentor (62.5% (n = 20) vs 48.1% (n = 39)), receiving at least one lecture from a urologist (62.5% (n = 20) vs 40.7% (n = 33)), and knowing an upperclassman interested in pursuing urology (56.2% (n = 18) vs 43.2% (n = 35)). Similarly, 65.6% (n = 21) of MD students reported knowing a peer in their same year who was interested in urology, compared with 58.0% (n = 47) of DO students (Figure 3). None of these differences were statistically significant, including the ability to find a urologist mentor (risk difference, 14.4%; 95% CI: -7.6% to 36.4%; p = 0.18), receiving at least one lecture from a urologist (risk difference, 21.8%; 95% CI: -0.8% to 44.4%; p = 0.06), knowing an upperclassman interested in urology (risk difference, 13.0%; 95% CI: -8.5% to 34.5%; p = 0.28), and knowing a peer in the same year interested in urology (risk difference, 7.6%; 95% CI: -14.3% to 29.5%; p = 0.53) (all Fisher’s exact tests).

Mentor availability

DO and MD students reported similar priorities when selecting mentors in urology. Most students in both groups rated having a mentor with the same degree as slightly or very important (70.4% (n = 57) DO vs 71.9% (n = 23) MD; risk difference, -1.5%; 95% CI: -20.5% to 17.5%; Fisher’s exact test, p = 0.86). Fewer respondents reported the importance of sharing the same gender identity with a mentor (38.3% (n = 31) DO vs 50.0% (n = 16) MD; risk difference, -11.7%; 95% CI: -32.0% to 8.6%; Fisher’s exact test, p = 0.27). The fewest respondents reported the importance of sharing the same racial or ethnic identity with a mentor (30.9% (n = 25) DO vs 21.9% (n = 7) MD; risk difference, 9.0%; 95% CI: -8.5% to 26.5%; Fisher’s exact test, p = 0.34).

Interest in urology 

In open-ended responses regarding interest in urology, students commonly reported the opportunity to engage in both surgical and outpatient practice and the perceived favorable lifestyle associated with the specialty. Additional reported reasons included interest in surgery, positive workplace culture within urology practices, perceived high compensation, opportunities for research advancement, interest in the scientific content of the specialty, and the ability to care for patients with urologic cancers.

In contrast, students who reported a lack of interest in urology commonly cited disinterest in the specialty’s scientific content, preference for another specialty, and the perceived competitiveness of the urology match. Additional reported reasons included limited familiarity with the specialty, discomfort with treating male sexual organs, lack of interest in surgical specialties, and greater interest in caring for other patient populations, such as women or children.

## Discussion

In this survey of osteopathic and allopathic medical students, DO students were less likely to perceive urology as a realistic career goal and reported limited exposure to the specialty, with nearly one-third indicating no exposure at all. Although differences in mentorship and research access did not reach statistical significance, MD students consistently reported greater availability of these resources. Students from both programs reported similar motivations for pursuing urology and valued having a mentor with the same degree background. Since both MD and DO groups valued degree-concordant mentorship, making osteopathic urologists more visible and accessible may be especially impactful. These findings suggest that differences in perceived feasibility may be associated with variations in exposure and institutional resources rather than motivation alone.

Taken together, these results point toward structural factors as potential contributors to differences in perceived feasibility. However, the observed between-group differences were accompanied by wide confidence intervals that included both clinically meaningful differences and no effect, reflecting substantial uncertainty in these estimates. As such, these findings should be interpreted cautiously and viewed as exploratory rather than definitive. In the context of national match disparities and limited osteopathic representation within urology, reduced representation may further influence perceptions of attainability.

To contextualize these findings, direct comparisons of DO and MD students’ perceptions of surgical specialties remain limited, as prior studies have largely focused on match outcomes, single-cohort analyses of osteopathic students, and program leadership perspectives. Wong and Mayrovitz reported that many osteopathic students lack familiarity with urology and have minimal preclinical interaction with urologists, while those considering the specialty strongly value early exposure and mentorship [10-14]. Although their study did not include MD comparisons, the present findings extend this work by demonstrating that differences in perceived feasibility and access to specialty-specific resources may exist between degree programs within the same institution. Importantly, the magnitude and direction of these differences were not precisely estimated, as reflected by the wide confidence intervals, which underscore the need for larger studies to better define the true effect size of these associations.

Program-level perspectives further support the importance of structured curricular exposure. Heard MA et al. found that urology residency programs cite sub-internships and research output as the main factors that make osteopathic applicants competitive [15,16]. Clinical immersion and scholarly work are not just helpful; they are expected. Similarly, Vogel AD et al. found that early exposure and mentorship are primary drivers of osteopathic student interest in cardiothoracic surgery, another highly competitive surgical field [17].

Building on these findings, disparities in early specialty exposure, mentorship access, and research opportunities may contribute not only to lower match rates but also to diminished perceptions of attainability among DO students pursuing competitive surgical careers. Although several comparisons did not reach statistical significance, the direction and magnitude of effect estimates were often consistent across domains, suggesting potentially meaningful differences despite limited statistical power. This uncertainty is further reflected in confidence intervals that were often wide and crossed the null, indicating that both meaningful differences and no difference remain plausible.

Improving equitable access to these structural supports represents a critical area for institutional intervention. Within osteopathic medical education, this may involve formalizing early specialty exposure, strengthening pathways to urology-specific research, and expanding mentorship networks, particularly those that facilitate degree-concordant mentorship. Notably, many DO students in our cohort expressed a desire for greater exposure despite limited access, reinforcing that interest in the specialty exists but may require institutional support to translate into sustained pursuit. Addressing these structural factors at the institutional level may represent an actionable strategy to improve perceived and actual competitiveness among osteopathic applicants.

To our knowledge, this study is the first to assess the perceptions of both DO and MD students at a single institution regarding matching into a surgical specialty. However, this study has several limitations. First, it was conducted at a single institution, which may limit generalizability to other medical schools with differing institutional affiliations and specialty exposure opportunities. The response rate was modest, particularly among DO students, raising the possibility of nonresponse bias, especially given the differential response rates between groups. Students who chose to participate may have had particularly high or low awareness of urology, stronger career uncertainty, or more pronounced views regarding institutional support. As such, observed differences between groups may partially reflect differences in response behavior rather than true underlying differences in attitudes or experiences.

To help mitigate this, the survey was distributed to all eligible students via institutional email lists, and participation was voluntary and anonymous to encourage broad and candid responses. Additionally, the relatively small sample size resulted in limited statistical power and wide confidence intervals around effect estimates, reducing the precision of between-group comparisons and increasing the likelihood of type II error. As a cross-sectional survey relying on self-reported data, the study is subject to recall and social desirability bias, and causal relationships between exposure, access to mentorship, and perceived feasibility cannot be established.

Finally, most respondents were preclinical students, which may not fully capture how perceptions evolve during clinical training or influence eventual residency application outcomes. Preclinical students often have limited exposure to surgical specialties, mentorship pathways, and residency competitiveness and therefore may not yet have a fully informed understanding of what constitutes a “realistic” career trajectory. As such, perceptions of urology as an attainable specialty in this cohort may reflect early impressions rather than decisions informed by clinical experience. This is supported by our stratified analysis, which demonstrated low perceived feasibility across both preclinical and clinical groups, although the small number of clinical respondents limits meaningful comparison. Accordingly, these findings should be interpreted as reflecting early-stage perceptions rather than application-stage judgments.

Future research should include multi-institutional studies to enhance generalizability and examine institutional factors that may influence interest in urology, such as the presence of affiliated urology residency programs and structured mentorship opportunities. Larger sample sizes will be critical to generate more precise effect estimates with narrower confidence intervals and to better distinguish true differences from random variation. Longitudinal studies following students throughout medical school may better characterize how perceptions of feasibility evolve over time with greater clinical exposure and influence application decisions. Additionally, intervention-based research evaluating the impact of targeted exposure, mentorship programs, and research access on urology application and match outcomes would help clarify the relationship between institutional resources and the successful pursuit of the specialty.

## Conclusions

In this single-institution survey comparing DO and MD students, DO students reported lower perceived feasibility of pursuing urology and reduced exposure to the specialty. While these differences were not statistically significant, the observed trends suggest that variation in access to mentorship, research opportunities, and early exposure may influence these perceptions. These findings are exploratory and highlight the need for larger, multi-institutional studies to better understand and address disparities in urology recruitment. Given persistent residency match rate disparities and the limited number of practicing osteopathic urologists, improving equitable access to early specialty exposure and mentorship may represent an actionable step toward strengthening osteopathic representation in surgical specialties.

## Appendices

Appendix 1 

**Table 2:** Survey questions and answer choices. The Survey Questions column contains all survey questions in the order in which they were presented. The Answer Choices column contains all answer choices available to respondents in the order in which they were presented.

Survey questions	Answer choices
In which program at Nova Southeastern University are you currently enrolled?	College of Osteopathic Medicine; College of Allopathic Medicine
What year of your medical education are you currently in?	M1; M2; M3; M4; Research year/Other
Which of the following best describes your gender?	Male; Female; Non-binary; Prefer not to say
Did you know that urology is listed as its own specialty in the match?	Yes; No
Have you considered pursuing urology as a specialty?	Yes, but decided not to pursue it; Yes, and will pursue it; No
Do you feel that matching into urology would be a realistic goal for you?	Yes; No; Not sure
Does your program encourage the pursuit of urology as a specialty?	Yes; No; Not sure
Do you have access to urology-specific research opportunities through your program?	Yes; No; Not sure
If you wanted to, do you think you could find a mentor in urology?	Yes; No; Not sure
How important is it to you to find a mentor who holds the degree you are pursuing (MD or DO)?	Not important; Slightly important; Very important
How important is it to you to find a mentor who identifies as the same gender as you?	Not important; Slightly important; Very important
How important is it to you to find a mentor who shares a similar racial or ethnic background?	Not important; Slightly important; Very important
Did you have exposure to urology prior to medical school?	Yes; No
Which of the following best describes your medical school experience?	I have had exposure to urology; I have not had exposure to urology, but I would like to; I have not had exposure to urology, and I would not like to
To your knowledge, have you received any lectures from a urologist during medical school?	Yes; No
Do you know any students in your class who are interested in urology?	Yes - one student; Yes - more than one student; No
Do you know any upperclassmen in your program who are interested in pursuing urology?	Yes - one student; Yes - more than one student; No
Does your program (MD or DO) have a club or organization for students who are interested in urology?	Yes; No; Not sure
If you are interested in pursuing urology as a specialty, what are your top three reasons, in order of importance? If not interested, skip.	Open-ended response
If you are not interested in pursuing urology as a specialty, what is the main reason?	Open-ended response
